# Supplementary material for: Splice-Junction-Based Mapping of Alternative Isoforms in the Human Proteome
Source: Cell Rep. Author manuscript; Available in PMC 2020 Jan 15. (PMC6961840; doi:10.1016/j.celrep.2019.11.026)
Supplement: 2 [file NIHMS1546469-supplement-2.zip › DF1/PXD010154/Spleen/Spleen_14_PDXDC1_QEVEATAGLLYVDDPNWSGIGVVSFRNSGR.pdf]

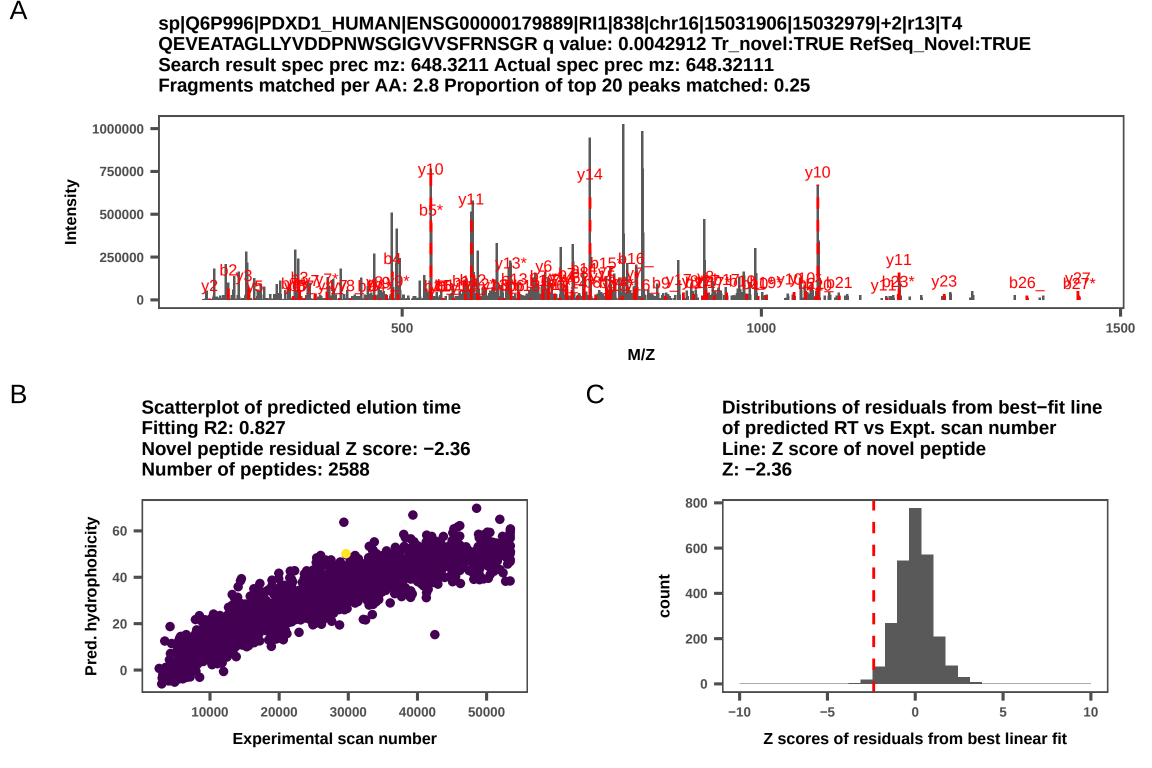
A
sp|Q6P996|PDXD1_HUMAN|ENSG00000179889|RI1|838|chr16|15031906|15032979|+2|r13|T4
QEVEATAGLLYVDDPNWSGIGVVSFRNSGR q value: 0.0042912 Tr_novel:TRUE RefSeq_Novel:TRUE
Search result spec prec mz: 648.3211 Actual spec prec mz: 648.32111
Fragments matched per AA: 2.8 Proportion of top 20 peaks matched: 0.25
Intensity
1000000
750000
500000
250000
0
500
1000
1500
M/Z
y10
b5*
y11
y14
y10
b4
y13*
y6
y11
y23
b26_
y2
b2
y3
y5
b21
B
Scatterplot of predicted elution time
Fitting R2: 0.827
Novel peptide residual Z score: −2.36
Number of peptides: 2588
Pred. hydrophobicity
60
40
20
0
10000
20000
30000
40000
50000
Experimental scan number
C
Distributions of residuals from best−fit line
of predicted RT vs Expt. scan number
Line: Z score of novel peptide
Z: −2.36
count
800
600
400
200
0
−10
−5
0
5
10
Z scores of residuals from best linear fit
